# Pre-Visual Detection of Pine Wilt Disease Using an Optimized PSRI Derived from Hyperspectral Drone Imagery

**DOI:** 10.3390/plants15172724

**Published:** 2026-09-05

**Authors:** Run Yu, He Weng, Dan Guo, Mingqing Weng, Ziyi You, Feiping Zhang, Songqing Wu

**Affiliations:** 1 State Key Laboratory of Agricultural and Forestry Biosecurity, College of Forestry, Fujian Agriculture and Forestry University, Fuzhou 350002, China; 2 Key Laboratory of Integrated Pest Management in Ecological Forests, Fujian Agriculture and Forestry University, Fuzhou 350002, China

**Keywords:** hyperspectral, multi-spectral, pine wilt disease, spectral features, early monitoring model

## Abstract

Pine wilt disease (PWD) is a devastating infectious disease of pine trees caused by the invasion of *Bursaphelenchus xylophilus*. Achieving rapid and accurate identification of pine trees in the early stages of infection is critical for preventing and controlling its spread, particularly for early warning and intervention before the plants exhibit obvious discoloration symptoms. The Plant Senescence Reflectance Index (PSRI) has shown significant potential for the early detection of PWD. However, existing studies often use its default band combinations for calculation, which may fail to fully exploit key wavelength information that is more sensitive to pre-symptomatic PWD stress. Based on unmanned aerial vehicle (UAV) hyperspectral imaging data, the present work systematically optimizes and reconstructs the three band parameters of the PSRI to explore an optimal wavelength combination more suitable for the early identification of PWD-infected trees. The results indicate that compared to the original settings of the PSRI, the wavelength combination of 490-666-700 nm performs better in pre-visually identifying infected pine trees in the early stages of infection, achieving a detection accuracy of 82.71%. Our work identifies an optimized PSRI wavelength combination more sensitive to the pre-visual early stage of PWD based on hyperspectral data. This method demonstrates promising potential for pre-visual detection of PWD before visible symptoms appear, which may provide an earlier opportunity for PWD monitoring and intervention.

## 1. Introduction

Pine wilt disease (PWD), caused by the pinewood nematode (*Bursaphelenchus xylophilus*) and primarily transmitted by *Monochamus* spp., is one of the most destructive diseases affecting pine forests [1]. Following infection, the nematode can rapidly disrupt water transport and physiological processes in host trees, resulting in progressive decline, crown discoloration, and eventually tree mortality [2]. Because susceptible pine trees may die within a relatively short period after infection, PWD can cause substantial damage at both individual-tree and stand scales and poses a serious threat to forest health and ecosystem stability [3]. PWD was first recognized as a major forest disease in East Asia and has subsequently spread across an increasing number of regions [3]. It has caused extensive damage in countries including Japan, China, and the Republic of Korea, and its occurrence has also been reported in parts of Europe [3,4]. In China, PWD has become one of the major threats to pine forest resources, with its distribution expanding across multiple provinces and regions [4,5,6]. The economic consequences of PWD are also substantial. In addition to the direct loss of timber resources caused by tree mortality, considerable resources are required for disease surveillance, quarantine, infected-tree removal, transportation and disposal, and vector control. Previous assessments have documented substantial economic losses associated with PWD outbreaks, indicating that the disease can impose a considerable financial burden on forest management and local forestry industries [5,6,7].

PWD develops rapidly, making timely and accurate early detection of infected trees a key aspect of prevention and control [2,3]. Especially at the early stage prior to the emergence of visible symptoms (i.e., pre-visual stage), timely identification of anomalous remote sensing (RS) signals alongside deployment of targeted prevention and control measures contribute greatly to curbing disease transmission, cutting forestry economic losses and elevating the overall operational efficiency of PWD management [4,5]. Due to its flexibility, non-invasiveness and high efficiency in data acquisition, unmanned aerial vehicle (UAV) RS has been widely applied in PWD detection [3,6,7,8,9,10]. RGB or multispectral RS is limited by its relatively wide band settings, and its spectral information usually represents an average response to signals within a certain wavelength range, making it difficult to capture the weak spectral features caused by subtle physiological and biochemical changes in the early stage of infection. Since the external symptoms of PWD are not obvious in the early stage, while internal physiological processes have already changed, these subtle changes may be reflected in specific narrow spectral regions or continuous spectral features, which can be more effectively characterized using hyperspectral data. Therefore, RGB and multispectral RS have certain limitations in extracting pre-symptomatic disease stress signals, leading to their relatively limited performance in early PWD detection [10,11,12,13,14].

Hyperspectral RS provides densely sampled spectral information across a broad wavelength range, which, depending on the sensor configuration and spectral range, can facilitate the characterization of subtle spectral responses associated with changes in physiological and biochemical characteristics during the early stages of disease infection. Compared with RGB and multispectral RS, it can identify characteristic wavelengths that are more sensitive to disease stress, particularly when these wavelengths are not adequately covered by the available RGB or multispectral bands, and thereby better exploit the potential information associated with early physiological changes in plants. This enhances the ability to perceive pre-symptomatic disease stress signals of PWD, achieving more accurate and reliable early detection [14,15]. Huang et al. found that the wavelengths sensitive to PWD are located at the green peak (538–580 nm), at the red-edge (665–779 nm), and near the water absorption feature in the SWIR region (1849–1862 nm) [16]. While the green and red-edge regions are covered by many multispectral sensors, the relatively dense spectral sampling of hyperspectral data may provide additional flexibility for identifying and optimizing disease-sensitive wavelengths within these regions. Through spectral unmixing analysis of hyperspectral images, Jeong et al. discovered that the spectral characteristics of sensitive endmembers in the early stages of PWD infection are mainly manifested as a significant increase in reflectance in the red regions (approximately 600–700 nm, which includes the chlorophyll absorption peak at about 680 nm) [17]. Similarly, Wu et al. found that there are significant spectral differences between healthy and infected pine trees in the wavelength range of 638–744 nm [7]. Zhang et al. used the Euclidean distance algorithm and found that the spectral ranges of 1974–2340 nm and 455–677nm are the optimal diagnostic windows [18]. Li et al. tested the most informative wavelengths for early detection and found that using the first derivative at 714 nm has stable performance in identifying PWD stress [14]. In another study, by identifying hyperspectral signals of *Pinus massoniana* under different stresses, Guo et al. found that PWD triggers responses in the blue-to-red-edge region (450–760 nm) [19]. As a result, it can be observed that there are significant differences in the optimal wavelength combinations recommended for early PWD detection in previous studies. And the standards have not yet been unified, lacking generalization and universality. This heterogeneity in wavelength selection creates operational decision complexity for forest managers in practical applications, making it difficult to determine which set of wavelength combinations is suitable for specific current needs.

Moreover, wavelength reflectance is susceptible to external factors such as lighting, atmospheric environment, observation angle, background elements and so on, exhibiting poor stability across different contexts and being prone to noise interference [7,20]. In contrast, vegetation indices (VIs) are constructed based on specific mathematical combinations of multiple wavelength reflectances, which can mitigate the influence of environmental factors and random noise, thereby exhibiting better stability and robustness [21,22]. In addition, VIs comprehensively use information features from different wavelengths. Compared to single-wavelength reflectance, they can more effectively enhance the expression of vegetation physiological status and structural characteristics, thus possessing stronger information representation capabilities and biological significance. In previous studies on early PWD detection, authors have extensively used various VIs to mine disease stress information, thereby enhancing the representation of early physiological changes in host trees. For instance, the Normalized Difference Vegetation Index (NDVI) and Normalized Difference Spectral Index (NDSI), which reflect vegetation vitality and canopy structure characteristics; the Normalized Difference Water Index (NDWI) and Shortwave Infrared Water Stress Index (SIWSI), which reflect vegetation water content; the Chlorophyll Index (CI), which is used to estimate chlorophyll content; indices for specific pigments such as the Pigment-Specific Normalized Difference (PSND) and Pigment Simple Ratio (PSR); and the Photochemical Reflectance Index (PRI) and Plant Senescence Reflectance Index (PSRI), which are sensitive to changes in carotenoids [22,23,24,25,26,27,28,29], have all been employed to extract pre-symptomatic disease stress signals of PWD.

However, most current studies tend to introduce a large number of VIs simultaneously and select the optimal combination through feature selection methods. While this approach can improve model performance, it also has certain limitations. On one hand, authors usually find strong information redundancy and correlations among different VIs, which increases model complexity. On the other hand, most studies focus more on improving model accuracy while paying insufficient attention to the physiological significance of vegetation indices and their connection to the mechanisms of disease occurrence. Hence, determining how to select key indices that possess both biological significance and disease sensitivity remains an important issue in the early detection of PWD. In contrast to other traditional VIs, the PSRI is sensitive to chlorophyll degradation and relative changes in carotenoids as it integrates information from red, green and near-infrared regions. It can effectively characterize plant senescence and stress processes [30,31]. Since early PWD infection is often accompanied by impaired photosynthesis, changes in pigment composition and physiological dysfunction, the PSRI is considered a relatively sensitive indicator for early stress response and is widely recommended in relevant studies for the early detection of PWD [9,32,33,34]. More importantly, in our previous studies, the PSRI has demonstrated good potential in the early detection of PWD [34,35]. Therefore, the present work focuses on the PSRI with the aim of further exploring its potential in the early detection of PWD.

PSRIs employ fixed combinations of characteristic wavelengths, with their design primarily based on empirical summaries of general vegetation senescence processes. Under conditions of different species and specific stress types (such as PWD infection), the optimal sensitive spectral wavelengths for their spectral response may not be entirely consistent. Particularly in the early stages of PWD, physiological changes within the plant are latent and complex. Also, the sensitivity of different spectral wavelengths to stress responses may vary. Fixed wavelength combinations may limit the index’s ability to characterize specific stress signals. Consequently, it is necessary to perform targeted optimization and reconstruction of the wavelength parameters of the PSRI within specific disease scenarios to enhance its responsiveness to subtle spectral changes during the early stages of PWD.

In this study, “pre-visual detection” refers to the discrimination of potentially PWD-infected trees from healthy trees before obvious crown discoloration becomes visually detectable. It should not be interpreted as a definitive diagnosis of infection at the individual-tree level. Because physiological responses to PWN infection can be subtle and may overlap with spectral variations caused by environmental and canopy-related factors, remote sensing-based detection at this stage is inherently associated with uncertainty. Therefore, the objective of this study is to investigate whether optimizing the wavelength configuration of the PSRI can improve the sensitivity to PWD-related spectral responses and provide an earlier indication of potential infection, rather than to establish a definitive diagnostic system.

It is important to note that the objective of this study is not to develop a new classification or detection algorithm for PWD, but to optimize the spectral configuration of the PSRI for PWD-specific pre-visual detection. The conventional PSRI uses a fixed combination of wavelengths originally designed to characterize plant senescence. However, the spectral responses associated with PWN infection may differ from those associated with general plant senescence, particularly during the pre-visual stage. Therefore, rather than directly adopting the conventional PSRI configuration, this study systematically explores alternative wavelength combinations to identify a PWD-sensitive spectral configuration and determine whether the optimized PSRI can improve the discrimination between healthy and PWD-infected pine trees before visible crown discoloration occurs. Thus, the methodological contribution of this study lies primarily in spectral-index optimization and wavelength selection, rather than in the development of a new machine-learning or deep-learning detection framework.

Therefore, the main objectives of the present work is to explore whether a more refined PSRI, optimized through wavelength combinations and targeted at early stress in PWD, exists prior to the onset of visual symptoms, which in turn achieves higher early detection accuracy.

## 2. Results

### 2.1. Optimal Wavelength Screening of PSRI

Based on the original construction idea for the PSRI, we adopted a univariate wavelength optimization strategy. Under the condition of keeping the other two wavelengths unchanged, each wavelength parameter was replaced and optimized separately to evaluate the sensitivity of different wavelengths to the early detection of PWD visual symptoms. The results showed that the optimal wavelength combination was 490-666-700 nm (Table 1). Compared with the 503-679-749 nm wavelength combination used in the sensor-compatible conventional PSRI, the optimized wavelength combination exhibited higher recognition ability in the early detection of PWD, indicating that the preset wavelength configuration in the classic PSRI is not the optimal choice for early stress signals of PWD.

**Figure 1:**
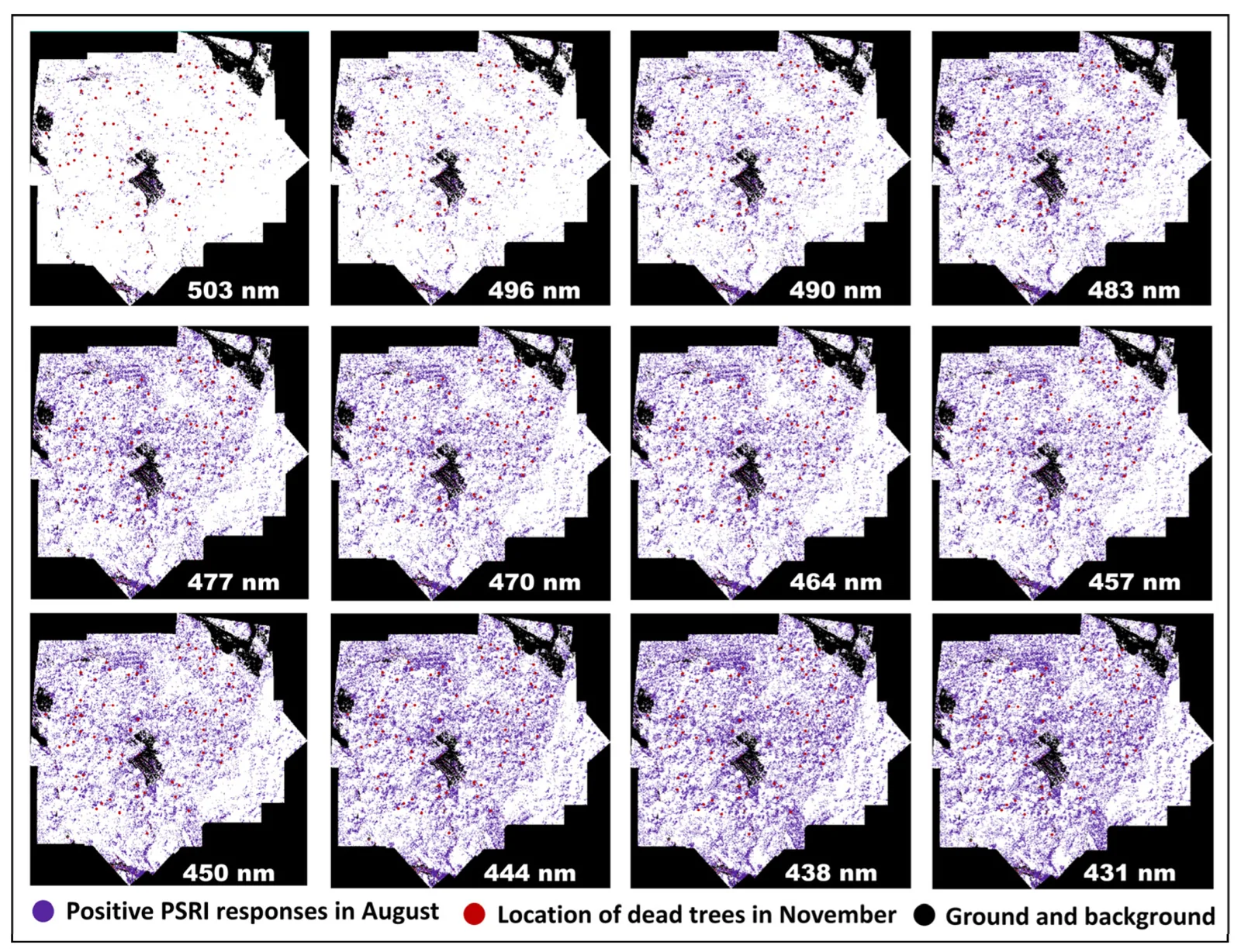
Detection for PWD-infected pine trees at pre-visual early stage (shown in purple) using different wavelength combinations. We kept 679 nm and 749 nm unchanged, and modified 503 nm.

**Figure 2:**
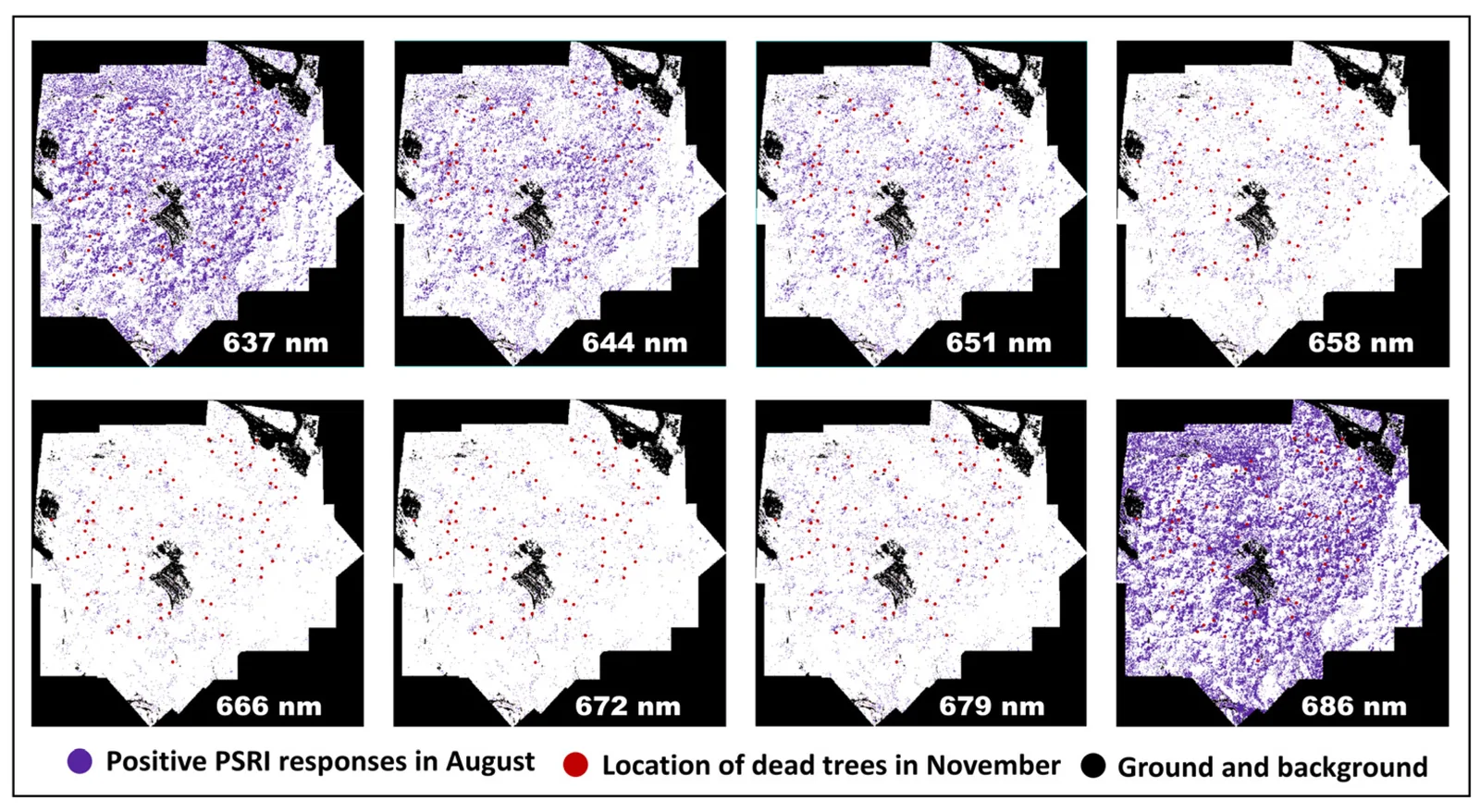
Detection for PWD-infected pine trees at pre-visual early stage (shown in purple) with different wavelength combinations. We kept 503 nm and 749 nm unchanged, and modified 679 nm.

**Figure 3:**
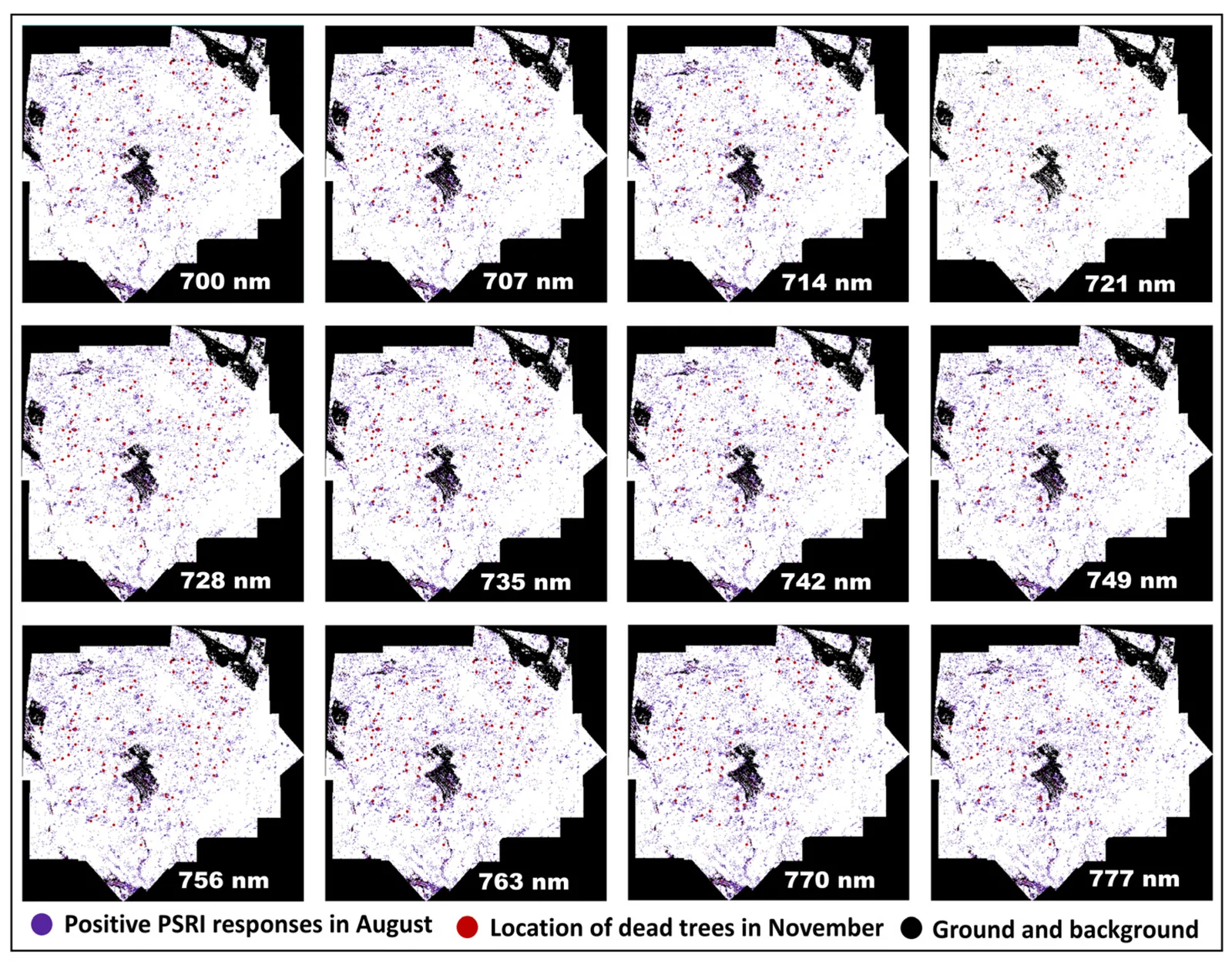
Detection for PWD-infected pine trees at pre-visual early stage (shown in purple) with different wavelength combinations. We kept 503 nm and 679 nm unchanged, and modified 749 nm.

Visualizations of the identification results obtained using different wavelength combinations are shown in Figure 1, Figure 2 and Figure 3. The spatial patterns of positive PSRI responses varied substantially with the selected wavelength combinations, indicating that the detection performance was sensitive to the choice of individual spectral components. Among the evaluated combinations, the PSRI configuration using approximately 490 nm in the blue region, 666 nm in the red region, and 700 nm in the red-edge region produced the most pronounced positive PSRI responses for pre-visually infected pine trees in August. Compared with the other wavelength combinations, this configuration generated a clearer and more spatially coherent distribution of positive response pixels within the crowns of trees that were subsequently confirmed to be diseased, suggesting a stronger spectral separation between pre-visually infected and healthy trees. The results indicate that the three optimized spectral components jointly enhanced the sensitivity of the PSRI to the subtle physiological changes associated with PWD before visible crown discoloration. Quantitative evaluation further confirmed the advantage of this optimized configuration, which achieved an overall accuracy of 82.71%, compared with 75.65% for the sensor-compatible conventional PSRI (Table 1).

### 2.2. Accuracy Evaluation

**Figure 4:**
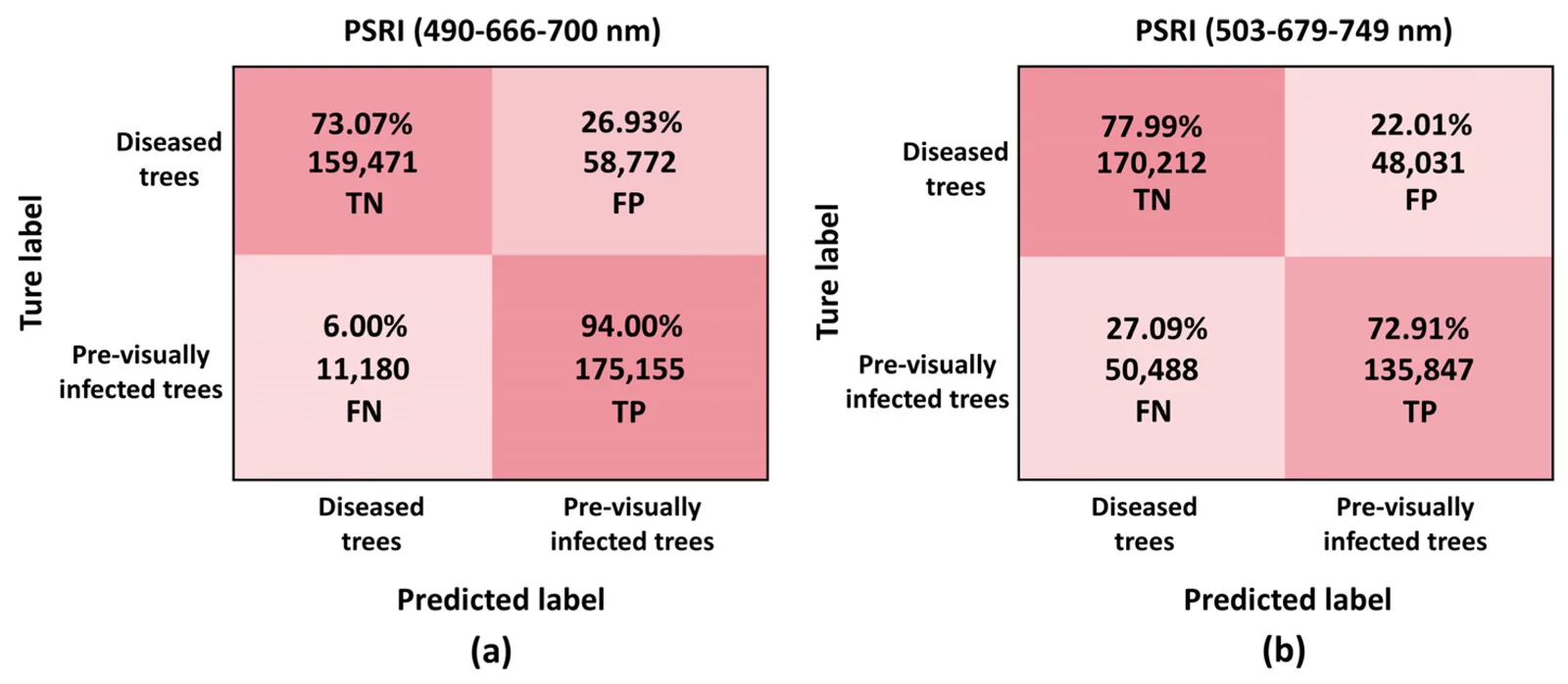
Pixel-level confusion matrices for the classification results using different PSRI wavelength combinations in August. (**a**) PSRI (490-666-700 nm); (**b**) PSRI (503-679-749 nm). TP: true positive; FP: false positive; TN: true negative; FN: false negative.

Figure 4 presents pixel-wise confusion matrices derived from two PSRI wavelength combinations. For PSRI (490-666-700 nm) (Figure 4a), misclassification of pre-visually infected tree pixels was limited (6.00%), whereas a proportion of diseased-tree pixels were incorrectly assigned to the pre-visually infected group. In comparison, PSRI (503-679-749 nm) (Figure 4b) produced substantial misclassification among pre-visually infected tree pixels (27.09%), even though misclassification within diseased-tree pixels was reduced. This discrepancy mainly originates from the spectral characteristics of pine trees at different infection stages. Pre-visually infected pines are in the early infection phase, with subtle spectral changes and no obvious crown discoloration. Small shifts in the selected wavelength ranges of the PSRI will alter its sensitivity to faint early-stage spectral signals. The band combination of 490-666-700 nm is more sensitive to the weak spectral variations induced by early infection, thus achieving better separation between the two categories. By contrast, the 503-679-749 nm combination is less responsive to these subtle early-infection signals, making it harder to distinguish pre-visually infected pixels from diseased ones and resulting in increased pixel-level confusion between the two classes. These observations demonstrate that careful band selection for the PSRI is critical for identifying early-stage pine infection at the pixel scale. Quantitative evaluation further confirmed the advantage of this optimized configuration, which achieved an overall accuracy of 82.71%, compared with 75.65% for the sensor-compatible conventional PSRI (Table 1). 

**Figure 5:**
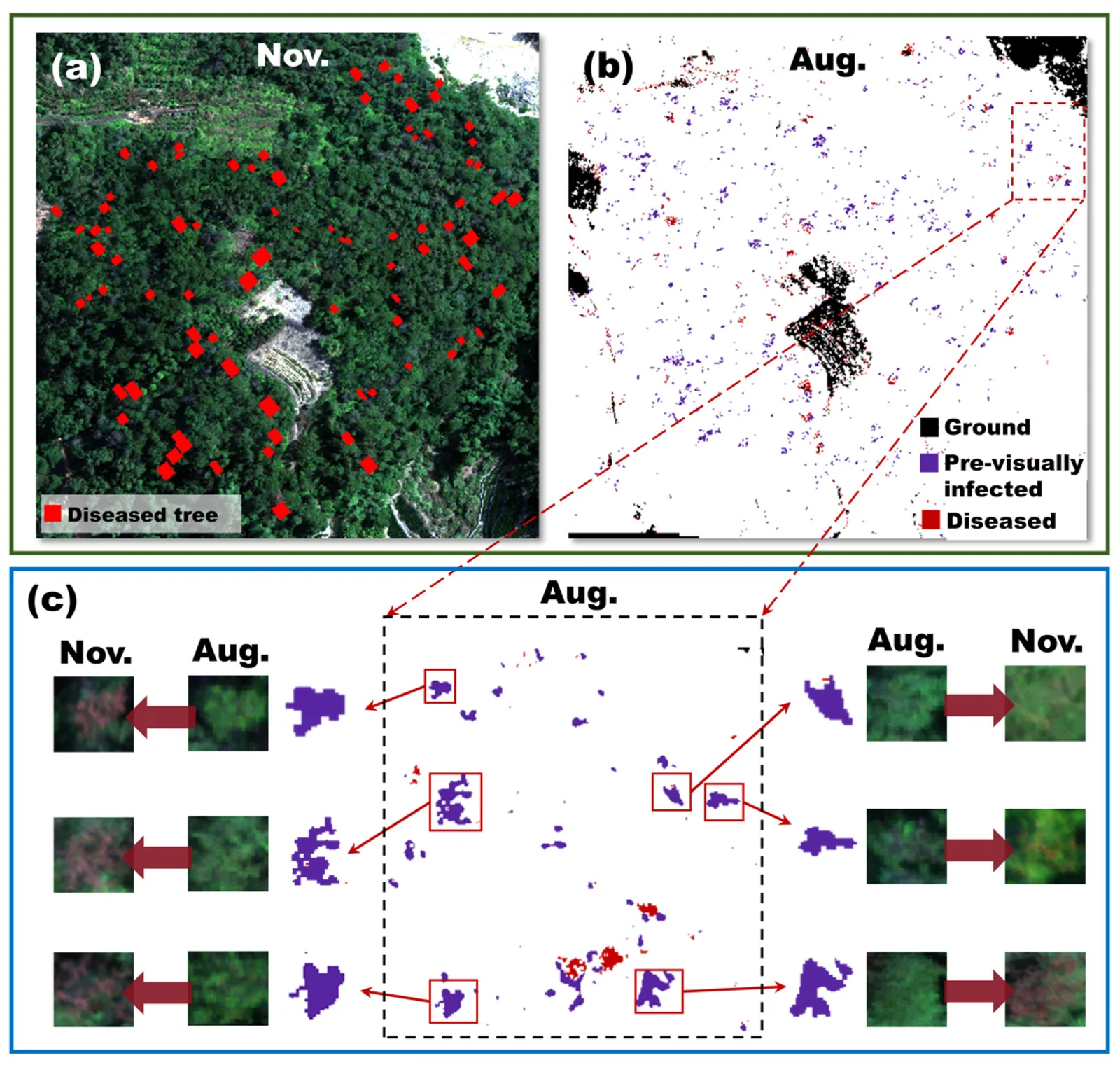
Discrimination map of PWD-infected trees using the optimal PSRI. (**a**) Condition of dead pine trees in November; (**b**) discrimination map of pre-visually infected trees in August, where purple areas represent discriminated early-stage infected trees and red areas represent diseased trees that were already diseased in August; (**c**) local magnified detail of the discrimination results of pre-visual early-stage infected trees.

In August, the optimized PSRI demonstrated a clear ability to identify anomalous canopy pixels associated with pine trees that subsequently developed visible symptoms of PWD, achieving a pixel-level identification accuracy of 82.71% (Figure 5). As defined in this study, pine trees that showed no visible crown discoloration in August but subsequently developed visible discoloration in November were considered to be in the pre-visual early stage of PWD. Figure 5a shows the condition of the trees confirmed as dead in November, while Figure 5b presents the corresponding discrimination results obtained from the optimized PSRI in August. The purple areas indicate canopy pixels identified as pre-visual PWD responses, whereas the red areas represent trees that had already exhibited visible disease symptoms in August. Notably, the positive PSRI responses were not randomly distributed across the study area but were mainly concentrated within the crowns of trees that subsequently developed visible symptoms in November. This spatial correspondence provides visual evidence that the optimized PSRI was able to capture subtle canopy spectral anomalies before visible discoloration became apparent. The locally magnified examples in Figure 5c further demonstrate that these pre-visual responses were spatially coherent within individual tree crowns rather than being restricted to isolated pixels. Together, these results indicate that the optimized wavelength combination enhanced the sensitivity of the PSRI to early PWD-related canopy changes and enabled the identification of potential infected trees during the pre-visual stage.

## 3. Discussion

### 3.1. Optimization of PSRI Wavelengths for PWD Detection

Through performing linear or non-linear combinations of different spectral wavelengths, VIs enhance the interpretability of RS imagery and mitigate the influence of external factors such as radiometric calibration errors, atmospheric water vapor interference, and illumination condition variations. As such, they hold significant theoretical importance and practical value in the efficient utilization of RS information [36]. The PSRI enhances sensitivity to changes in the ratio of carotenoids to chlorophyll and has been considered one of the more promising vegetation indices for early detection of PWD in previous studies [34,35]. Based on the original PSRI, this study systematically reconstructed and optimized its three wavelength parameters, yielding an optimal wavelength combination of 490-666-700 nm. Compared to the 503-679-749 nm band combination used in the original PSRI, the optimized index exhibited higher classification accuracy and discrimination ability in early PWD identification, indicating that the preset wavelength configuration in the classic PSRI is not the optimal solution for early PWD stress signals. The present work further illustrates that the spectral responses corresponding to different disease types and stress stages exhibit significant differences. Targeted optimization of VIs for specific disease scenarios is expected to significantly enhance their ability to characterize early physiological anomaly signals. On this basis, the optimized PSRI constructed in this study can more effectively capture subtle spectral changes prior to the appearance of PWD visual symptoms, providing a new technical pathway for early PWD monitoring. Notably, both the optimized red-light wavelength (666 nm) and the near-infrared transition wavelength (700 nm) shifted towards the red-edge region, suggesting that early PWD stress information may be more concentrated in the vicinity of the chlorophyll strong absorption zone and the red-edge transition regions. This characteristic is consistent with physiological processes such as disease-induced chlorophyll content reduction and impaired photosynthesis, supporting the rationality of the wavelength optimization results at the mechanistic level [37,38].

### 3.2. Selection of Classification Method

In terms of classification methods, decision trees are a widely used class of supervised classification algorithms, with classic representatives including ID3 and CART (Classification and Regression Tree), C4.5 [39,40]. This class of methods achieves classification decisions through hierarchical partitioning of the feature space, enabling effective representation of the nonlinear features of hyperspectral data. Concurrently, they offer high computational efficiency and relatively low demands on sample size and computing power, leading to their extensive application in remote sensing image classification. The C4.5 algorithm, building upon ID3, introduces a pruning strategy and utilizes the information gain ratio to select the optimal splitting attribute, thereby generating a decision tree model with a more concise structure. Compared to other classification methods, C4.5 not only achieves high classification accuracy but also produces rules with good interpretability. Accordingly, this study selects the decision tree framework and employs the C4.5 algorithm as the classifier for experimental analysis [41].

### 3.3. The Accuracy of Pre-Visual Detection of PWD

In many PWD remote sensing studies, the term “early detection” encompasses trees that have already entered an early symptomatic stage, in which physiological deterioration has begun to produce visible changes in needle color or canopy appearance. However, it is worth noting that even among studies focusing on trees that had already developed mild crown discoloration, a considerable number of studies reported detection accuracies below 80% [38]. When the comparison is further restricted to the more challenging pre-visual stage, performance is generally even lower. As summarized in Yu et al. [38], the detection accuracies reported by most studies targeting pre-visual PWD are below 80%. For example, Pan et al. [4] reported an accuracy of 50% for pre-visual PWD detection. Shi et al. [9] combined spectral and texture features, yet achieved an accuracy of only 62.5% at the pre-visual stage. Similarly, Yu et al. [34] reported a detection accuracy of 77% for pine trees that had not yet developed obvious crown discoloration. These studies indicate that accurate PWD discrimination before the emergence of conspicuous visual symptoms remains a highly challenging task, largely because the spectral and canopy structural changes induced by PWN infection are relatively subtle at this stage. These results clearly illustrate that detection performance is strongly dependent on disease stage and that detecting PWD before conspicuous visual symptoms emerge is considerably more challenging than detecting trees with established discoloration.

### 3.4. Limitations and Future Perspectives

#### 3.4.1. Need for Systematic Benchmarking and Comparative Evaluation

Despite the promising performance of the optimized PSRI, several limitations should be acknowledged. First, the present study primarily focused on optimizing the wavelength configuration of the PSRI rather than developing or benchmarking a new PWD detection algorithm. Therefore, the optimized PSRI was not comprehensively compared with a broad range of existing PWD detection methodologies, particularly machine-learning- and deep-learning-based approaches. Such a comparison is potentially valuable for further evaluating the practical advantage of the optimized spectral index.

However, a rigorous comparison should ideally be conducted under a unified experimental framework, because the performance reported by existing PWD detection studies is often influenced by differences in sensors, spectral resolution, spatial resolution, datasets, disease stages, feature extraction procedures, and evaluation protocols. Directly comparing reported accuracy values across different studies may therefore not provide a fair assessment of methodological superiority.

Future studies should address this issue by establishing a common benchmark dataset and standardized evaluation protocol, and by systematically comparing the optimized PSRI with conventional vegetation indices, alternative spectral indices, machine-learning models, and deep-learning-based detection approaches. Such a controlled comparison would help determine whether the improved spectral sensitivity achieved through wavelength optimization can be translated into a consistent advantage in practical PWD detection and early-warning applications. In addition, validation across different pine species, geographic regions, acquisition conditions, and independent field datasets will be necessary to further assess the robustness and generalizability of the optimized PSRI.

#### 3.4.2. Generalizability and Transferability Across Different Conditions

Another important limitation of this study is the lack of independent multi-site datasets for evaluating the generalizability of the optimized wavelength combination. The optimal wavelengths were identified and evaluated using data collected under the conditions of the present study, and independent datasets from other geographic regions were not available for external validation in the current work. Consequently, we cannot yet determine whether the optimized PSRI maintains the same wavelength configuration and detection performance across different pine species, geographic locations, canopy structures, or environmental conditions.

This issue is particularly important for practical PWD monitoring because spectral responses measured by UAV platforms can be affected by multiple sources of variation. Differences in pine species and physiological characteristics may alter the spectral response to PWN infection, while geographic and environmental factors, including temperature, precipitation, soil and understory background, illumination conditions, and canopy structure, may further influence canopy reflectance. These factors could potentially affect the optimal wavelength configuration of the PSRI and should therefore be considered when assessing its transferability.

Future research will place particular emphasis on independent multi-site and multi-condition validation of the optimized PSRI. Specifically, datasets from different geographic regions, pine species, stand structures, and environmental conditions will be collected and evaluated using a consistent experimental and analytical framework. Such validation will allow us to determine whether the optimized wavelengths are stable across different conditions or whether site- or species-specific wavelength adjustments are necessary. Furthermore, identifying the major factors responsible for potential shifts in the optimal wavelength configuration will help clarify the physiological and environmental mechanisms underlying PSRI optimization and ultimately improve the robustness and practical applicability of the index for large-scale PWD early-warning applications.

Overall, the optimized PSRI demonstrated promising capability for discriminating potentially PWD-infected pine trees before obvious crown discoloration, achieving an accuracy of 82.7% under the conditions investigated in this study. These results suggest that wavelength optimization can improve the sensitivity of the PSRI to PWD-related spectral responses and may provide an earlier screening signal than visual assessment. However, the optimized PSRI should not be regarded as a definitive diagnostic tool, and its performance may be influenced by disease progression, environmental conditions, canopy structure, pine species, and other sources of spectral variability. Further independent validation across different sites and environmental conditions, together with complementary field-based diagnosis, is required before the method can be considered for broader operational deployment.

#### 3.4.3. Temporal Resolution and Symptom-Onset Characterization

Another limitation of the present study is the limited temporal information available for symptom confirmation. Although the remote sensing observations provided information on the spectral responses preceding visible symptoms, systematic symptom assessment was available only at the November time point. Consequently, we could not determine whether some trees developed visible symptoms before November or whether other infected trees remained asymptomatic and developed visible symptoms after November. A more comprehensive temporal sampling design, including systematic symptom assessments before, during, and after the reference time point, would provide a stronger framework for determining the timing of symptom onset and for evaluating the temporal stability of pre-visual PWD detection. Such a design would also allow the performance of the optimized PSRI to be examined across trees with different disease progression rates and different symptom-emergence times.

Future studies will therefore incorporate multi-temporal symptom monitoring throughout the disease progression period, rather than relying on a single symptom-confirmation time point. Combining repeated UAV observations with continuous field symptom assessments would allow the onset of visible symptoms to be more precisely determined for individual trees and would provide a more robust basis for evaluating whether the optimized PSRI can consistently identify PWD at different stages before visible symptoms emerge.

#### 3.4.4. Limitations of the Wavelength Optimization Strategy

Although the component-wise optimization adopted in this study provides an interpretable approach for evaluating the sensitivity of individual PSRI components, it does not exhaustively explore all possible combinations of the three wavelengths. In principle, simultaneously varying all three wavelengths could identify spectral combinations that are not captured by the one-at-a-time optimization strategy and may therefore provide a more comprehensive assessment of the global wavelength space. Previous hyperspectral studies have used exhaustive searches of two- or three-band combinations to identify optimal spectral indices for specific physiological traits or disease conditions.

However, an exhaustive three-dimensional search would substantially increase the number of candidate wavelength combinations. More importantly, selecting the single combination with the highest performance from a very large search space may increase the risk of obtaining a dataset-specific empirical optimum, particularly when the number of independent field samples is limited. Therefore, the optimal combination identified through exhaustive searching would ideally require independent validation or nested cross-validation to determine whether its performance is robust and transferable. In the present study, we therefore focused on a targeted optimization of the established PSRI structure rather than claiming that the resulting wavelength combination represents a global optimum across the entire spectral space.

Another potential improvement is to replace individual wavelengths with wavelength intervals and calculate the mean reflectance within each interval. Such an approach may reduce the influence of noise and small fluctuations at individual wavelengths and could potentially improve the robustness of the index across different sensors and acquisition conditions. Nevertheless, the primary objective of the present study was to identify specific spectral locations that were most sensitive to PWD-related physiological changes. Averaging reflectance over a relatively broad wavelength interval may smooth out narrow spectral features or spectral shifts associated with pigment absorption and red-edge responses, potentially reducing the spectral specificity that is of particular interest in the present exploratory analysis. Therefore, individual wavelengths were retained in the current study to preserve the spectral sensitivity of each PSRI component. Future research should compare single-wavelength and interval-based formulations to determine whether spectral averaging can improve the robustness and transferability of the optimized PSRI.

Future studies should further evaluate the optimized PSRI using a more comprehensive wavelength-search framework, including simultaneous three-wavelength optimization and interval-based spectral averaging. Such analyses, combined with independent datasets from different geographic regions, pine species, and environmental conditions, would help determine whether the identified spectral region represents a stable physiological signal associated with PWD or a dataset-specific optimum.

## 4. Materials and Methods

### 4.1. Study Area

**Figure 6:**
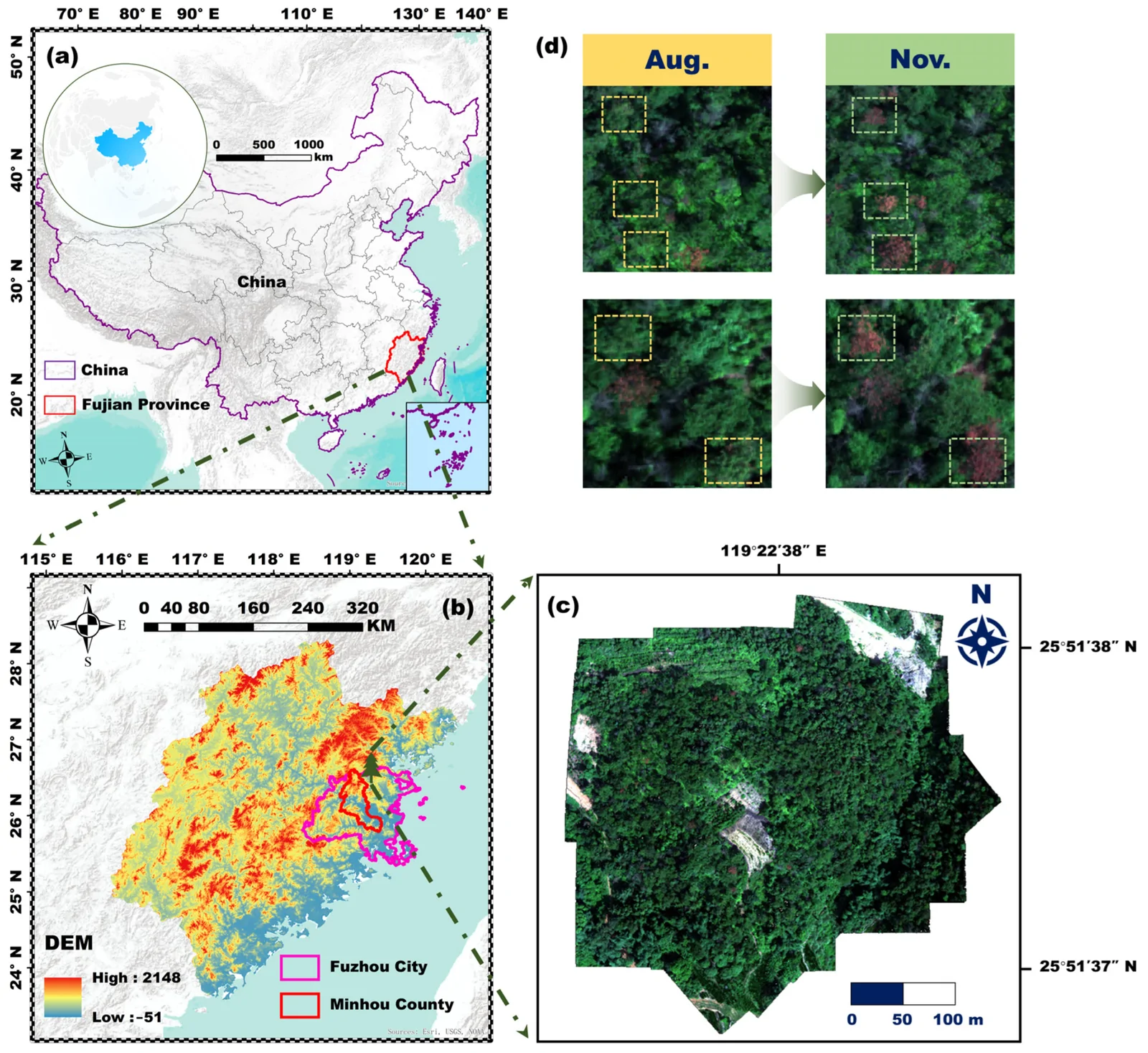
Schematic diagram of the sample plot. (**a**) Location of Fujian Province, China; (**b**) location of Minhou County, Fuzhou City, Fujian Province; (**c**) UAV hyperspectral image of the sample plot (acquired in August, displayed in RGB bands); (**d**) schematic diagram of the process of pine trees changing color from never discolored (in August) to discolored (in November).

The study was conducted in Qingkou Town, Minhou County, Fuzhou City, Fujian Province, China (119°34′40″ E, 25°85′92″ N) (Figure 6). Fujian Province is located in Southeastern China, a coastal province located along the southeast coast of mainland China. The study area is situated within a subtropical region characterized by a warm and humid climate and extensive forest resources, providing suitable conditions for the occurrence and development of PWD. The forest stand in the plot is dominated by *Pinus massoniana*, with no discolored broad-leaved trees. The total area covered by the field experiment was approximately 0.1 km^2^. The field investigation and image acquisition were conducted from August to November 2020.

### 4.2. UAV-Based Hyperspectral Image Acquisition and Processing

Hyperspectral images were acquired using a UAV DJI M300 (DJI, Shenzhen, China) equipped with a GaiaSky-mini2-VN hyperspectral imaging system (Dualix Spectral Imaging, Wuxi, China). Image acquisition was conducted in the field on 20 August and 20 November 2020. Flights were performed under relatively stable illumination conditions to minimize variations in incident solar radiation and associated effects on canopy reflectance. The UAV flight altitude was set to 200 m, and the flight speed was maintained at 3 m/s. To achieve reflectance correction, three white standard cloths measuring 1.8 m × 2.0 m were laid out in an unobstructed open plot as references. Before each flight, the imaging system was appropriately configured and radiometrically calibrated according to the manufacturer’s specifications. During image acquisition, the UAV followed pre-designed flight routes with sufficient forward (70%) and side overlap (70%) between adjacent images to ensure complete coverage of the experimental area and facilitate subsequent image mosaicking and geometric correction. Images were acquired from an approximately nadir view to minimize geometric distortion and differences in canopy viewing geometry.

Following acquisition, the raw images were subjected to the preprocessing procedures described below, including radiometric correction, geometric correction, image registration, orthomosaic generation, and reflectance normalization [35]. The resulting imagery had a ground sampling distance (GSD) of approximately 6 cm/pixel. The processed imagery was subsequently used to extract the spectral information required for PSRI calculation and wavelength optimization.

### 4.3. Definition and Sample Selection of Early-Disease Pines

Early detection of diseased pine trees before visual symptoms appear can provide more response time for PWD management, thus having significant implications for reducing the risk of disease spread and minimizing forest loss. Hence, the present work focuses on pine trees in the early-disease stage before the appearance of visual symptoms, and does not analyze diseased trees that have already exhibited obvious discoloration. To define diseased pine trees in the pre-visual early stage, the present work identified pine trees with canopy discoloration in November and retrospectively determined their crown plots in August, when visual symptoms had not yet appeared, as early-disease samples for subsequent hyperspectral data collection and analysis (Figure 6d). The selection of samples of early-disease pine trees was performed using the ROI tool in ENVI 5.3 (Harris Corporation, Melbourne, FL, USA). A total of 95 pre-visually infected pine trees (crowns remained un-discolored in August but developed discoloration by November) and 96 diseased pine trees (already discolored by August) were selected for subsequent analysis.

In this study, November 2020 was used as the reference time point for identifying trees that had developed severe visible symptoms or had died. This time point was selected based on the field investigation schedule and the availability of systematic symptom-assessment data. Importantly, the November assessment was used as a temporal reference for defining the progression of PWD symptoms, rather than implying that November represents a universal or fixed time point for PWD symptom development. Because systematic and comparable symptom-assessment data were only available for November in the present dataset, additional symptom-confirmation time points before and after November were not available for inclusion in the current analysis. Therefore, the present study cannot directly determine whether individual trees developed visible symptoms earlier or later than the November assessment.

### 4.4. Optimal Wavelength Screening

By modifying the wavelengths in the original PSRI formula and evaluating the detection accuracy, the optimal wavelength for early PWD detection was screened. More precisely, the wavelengths of R_λi_, R_λj_ and R_λk_ in the following formula were adjusted as new inputs.

*PSRI* = (*R_λi_* − *R_λj_*)/*R_λk_*

where *R_λi_* is the reflectance value at wavelength *λi*. In the formula of the original PSRI, *R_λi_*, *R_λj_* and *R_λk_* are, respectively, 680 nm, 500 nm and 750 nm. Because the exact nominal wavelengths of the conventional PSRI (500, 678, and 750 nm) were not available in the hyperspectral dataset, the nearest available bands, namely 503, 679, and 749 nm, were used to construct a sensor-compatible conventional PSRI. Here, 503–679–749 nm should be regarded as the sensor-compatible implementation of the conventional PSRI rather than an exact reproduction of its nominal wavelength configuration.

The objective of the wavelength optimization in this study was to determine whether the conventional wavelength configuration of the PSRI could be adjusted to improve its sensitivity to the subtle spectral responses associated with pre-visual PWD, while retaining the original three-band structure and physiological interpretability of the index. Therefore, rather than developing an unconstrained three-band spectral index through an exhaustive search of the entire spectral space, we adopted a component-wise wavelength sensitivity analysis. Specifically, two wavelength components were retained at their original positions while the third wavelength was systematically varied within the available spectral range. This procedure was repeated for each of the three PSRI components, allowing the sensitivity of individual wavelength components to PWD-related spectral responses to be evaluated independently [42].

### 4.5. Accuracy Assessment

In this study, the dataset was divided into a training set (70%) and a testing set (30%), and we used Classifier 4.5 (C4.5) algorithm to construct a classification model. C4.5 is an extension of the Iterative Dichotomiser 3 (ID3) algorithm used for generating decision trees for classification tasks.

We employ overall accuracy and the Kappa coefficient to evaluate detection precision. OA refers to the ratio of correctly classified pixels to the total number of categories, which is equal to the sum of correctly classified pixels divided by the total number of pixels [43]. The calculation process can be expressed by the following formula:

*OA* = (*TP* + *TN*)/(*TP* + *FN* + *FP* + *TN*)

where *TP* is true positive, the number of pixels that are early-disease vegetation pixels and are also detected as early-disease vegetation pixels. *FP* is false positive, which is the number of pixels that are early-disease vegetation pixels but are detected as non-early-disease vegetation pixels. *TN* is true negative, which is the number of pixels that are other ground features and are also detected as other ground features. *FN* is false negative, which is the number of pixels that are other ground features but are detected as early-disease vegetation pixels.

The Kappa coefficient is a proportion that represents the proportion of reduction in errors compared to a classification made by chance [44], and its calculation process can be expressed by the following formula:

*Kappa* = (*OA* − *eAccuracy*)/(1 − *eAccuracy*)

$$\mathrm{eAccuracy}=(\sum_{i=1}^{n}\mathrm{Vp}\times \mathrm{Vm})/S^{2}$$

where *OA* is the overall accuracy, *n* is the number of classes, *Vp* is the predicted value, *Vm* is the measured value, and *S* is the sample number.

## 5. Conclusions

Built upon UAV hyperspectral imaging data, we reconstruct and optimize the wavelength structure of the PSRI for detecting early stress signals of PWD. Optimized wavelength combinations (490-666-700 nm) with enhanced sensitivity were obtained. The results indicate that compared to the original PSRI parameter, the optimized index can more effectively characterize subtle spectral variations prior to the appearance of visual symptoms, significantly improving the identification capability for early-infected pine trees. Further investigation reveals the crucial role of the red-edge transition regions in the early response to PWD, validating the necessity and feasibility of optimizing VI parameters for specific disease contexts. The optimized PSRI demonstrated improved capability for pre-visual discrimination of PWD under the experimental conditions investigated in this study. However, its generalizability across different pine species, geographic regions, canopy structures, and environmental conditions remains to be validated. Multi-site and independent-dataset validation will therefore be an important step toward establishing the robustness and practical applicability of the optimized PSRI for broader PWD monitoring and early-warning applications.

## Figures and Tables

**Table 1:** Precision comparison of different wavelength combinations.

Constant Wavelength	Modified Wavelength (nm)	Overall Accuracy (OA)	Kappa Coefficient (Kappa)
Kept 679 nm and 749 nm unchanged, and modified 503 nm	679-503-749	75.65%	0.4779
	679-497-749	79.78%	0.5864
	679-490-749	80.50%	0.6131
	679-484-749	79.93%	0.607
	679-477-749	79.96%	0.6082
	679-471-749	79.66%	0.6013
	679-464-749	79.33%	0.5938
	679-457-749	78.99%	0.587
	679-451-749	78.28%	0.576
	679-445-749	76.54%	0.5489
	679-438-749	73.54%	0.5037
	679-432-749	69.69%	0.4444
Kept 503 nm and 749 nm unchanged, and modified 679 nm	638-503-749	48.41%	0.1706
	645-503-749	58.20%	0.2994
	652-503-749	72.57%	0.5006
	659-503-749	78.19%	0.5803
	666-503-749	79.46%	0.5777
	672-503-749	76.05%	0.4882
	679-503-749	78.43%	0.5501
	686-503-749	55.83%	0.2673
Kept 503 nm and 679 nm unchanged, and modified 749 nm	679-503-693	75.86%	0.5032
	679-503-700	77.30%	0.5233
	679-503-707	77.03%	0.5155
	679-503-714	76.74%	0.5075
	679-503-721	76.43%	0.4991
	679-503-728	76.13%	0.4908
	679-503-735	75.92%	0.4851
	679-503-742	75.76%	0.4808
	679-503-749 (the sensor-compatible conventional PSRI)	75.65%	0.4779
	679-503-757	75.60%	0.4764
	679-503-764	75.52%	0.4746
	679-503-771	75.52%	0.4745
	679-503-778	75.51%	0.4742
Optimized PSRI	503-666-700	82.71%	0.6225

## Data Availability

The raw data supporting the conclusions of this article will be made available by the authors on request.
